# An Enzyme from *Aristolochia indica* Destabilizes Fibrin-β Amyloid Co-Aggregate: Implication in Cerebrovascular Diseases

**DOI:** 10.1371/journal.pone.0141986

**Published:** 2015-11-06

**Authors:** Payel Bhattacharjee, Debasish Bhattacharyya

**Affiliations:** Division of Structural Biology and Bioinformatics, CSIR -Indian Institute of Chemical Biology, Jadavpur, Kolkata, 700032, India; Aligarh Muslim University, INDIA

## Abstract

Fibrinogen and β-amyloid (Aβ) peptide independently form ordered aggregates but in combination, they form disordered structures which are resistant to fibrinolytic enzymes like plasmin and cause severity in cerebral amyloid angiopathy (CAA). A novel enzyme of 31.3 kDa has been isolated from the root of the medicinal plant *Aristolochia indica* that showed fibrinolytic as well as fibrin-Aβ co-aggregate destabilizing properties. This enzyme is functionally distinct from plasmin. Thrombolytic action of the enzyme was demonstrated in rat model. The potency of the plant enzyme in degrading fibrin and fibrin-plasma protein (Aβ, human serum albumin, lysozyme, transthyretin and fibronectin) co-aggregates was demonstrated by atomic force microscopy, scanning electron microscopy and confocal microscopy that showed better potency of the plant enzyme as compared to plasmin. Moreover, the plant enzyme inhibited localization of the co-aggregate inside SH-SY5Y human neuroblastoma cells and also co-aggregate induced cytotoxicity. Plasmin was inefficient in this respect. In the background of limited options for fragmentation of these co-aggregates, the plant enzyme may appear as a potential proteolytic enzyme.

## Introduction

Fibrin clot formation is often accompanied by β-amyloid (Aβ) peptide aggregates leading to severity in cerebrovascular damage in cerebral amyloid angiopathy (CAA) [1–3]. With increasing age, due to impairment of perivascular drainage, Aβ peptides accumulate in the cerebrovascular basement membrane leading to leukoaraiosis and inflammatory changes in CAA patients [4–6]. It may be an independent disease but is often accompanied by Alzheimer’s disease (AD) making elucidation of the patho-mechanism more complicated. Under normal physiological conditions, thrombosis and thrombolysis are balanced processes which are mediated by plasmin, a serine protease that cleaves the fibrin network at specific sites [7]. Fibrin itself regulates the formation of plasmin from plasminogen by tissue plasminogen activator (tPA). Once fibrinogen is converted to fibrin, Aα148–160 and γ312–324 sequences of fibrinogen become available for binding to tPA and plasminogen, which are critical for efficient fibrinolysis [8,9]. Plasmin also plays critical role in degradation of Aβ and its clearance from brain [10]. Plasmin is activated by the accumulation of Aβ peptide *via* tPA and reduces Aβ burden in the brain of APP/PS1 transgenic mice by degrading Aβ monomer and oligomers [11].

Fibrinolysis and degradation of Aβ aggregates are affected if the aggregates are heterogeneous *i*.*e*. constituted of other adhering proteins. The bound proteins may sterically protect the hydrolysable bonds of fibrin from the action of plasmin. Aβ peptide alters the structure of fibrin upon binding leading to the formation of lysis resistant clots. There are two proposed mechanisms of Aβ mediated delayed lysis of clots. One states that Aβ binds to fibrinogen β-chain near the β-hole, which is in close proximity to the Aα148–160 sequences of fibrin and obstructs the binding of plasminogen to fibrin. Alternately, Aβ alters the porosity of fibrin clot. Several studies showed correlation between pore size along with diameter of fiber and fibrinolysis. Tight fibrin network composed of small pores and high fiber density is less efficiently degraded by plasmin than a loose network composed of thick fibers [2,12,13]. Under normal conditions, the concentration of fibrinogen in blood is 2–4 mg/dL. It circulates through the brain and spinal cord vasculature without entering into the central nervous system (CNS) due to blood brain barrier (BBB). BBB creates a permeability barrier between brain capillaries and the extracellular fluid in brain [14]. However, in pathological conditions like injury or diseases associated with vascular disruption, infection or inflammation of cerebrovascular region occurs for which the concentration of fibrinogen increases beyond the normal limit and the protein enters into CNS through disrupted BBB [15]. The synergistic effect of higher fibrinogen content and presence of Aβ peptide produce lysis resistant clots in neurovascular diseases which attributes to vascular deficiencies causing decreased blood flow, increased inflammation and neuronal death [1,16]. Lysis resistance is attributed to the inaccessibility of plasmin to the hydrolysable bonds. Fibrin-Aβ co-aggregate so formed has a denser network than the fibrin clot or Aβ aggregate; this causes steric hindrance to plasmin [13].

‘Boston criteria’ that combines clinical, neuroimaging and pathological parameters helps to overcome the difficulty of diagnosis for CAA which requires autopsy of sample. On the other hand, due to lack of specific treatment, CAA patients receive indirect therapy like administration of anticoagulants or drugs that are used for the treatment of AD. In fact, anticoagulants like warfarin increases mortality after intracerebral haemorrhage (ICH) and may not be safe for CAA patients [17,18]. Moreover, the drugs for AD are either cholinesterase inhibitors or NMDA receptor antagonists, which only help in symptomatic treatments. They do not treat the underlying problems associated with plaque or neurofibrillary tangle formation and neuronal damages [19]. Recently RU-505 has been identified as an inhibitor that target Aβ–fibrinogen interaction and significantly improved cognitive function of the AD transgenic mouse by inhibiting vessel occlusion and reducing vascular amyloid deposition in the cortical region of mouse brain [20]. Except RU-505, to our knowledge there is no other report on destabilization of fibrin-Aβ co-aggregate. Therefore, it is necessary to search for novel therapeutic agents that could destabilize fibrin clots together with fibrin-Aβ disordered co-aggregates.

*Aristolochia indica*, a medicinal plant of Indian subcontinent, is traditionally used for curing varieties of ailments. Enzymatic properties of the aqueous extract of its root have been characterized. It showed strong gelatinolytic, collagenase, nuclease, peroxidase and L- amino acid oxidase inhibition properties but did not show detectable hydrolytic activity with azocasein, azoalbumin, Nα-Benzoyl-L-arginine ethyl ester (BAEE) and Nα-p-Tosyl-L-arginine methyl ester (TAME) as substrate. Moreover, the aqueous extract did not show detectable toxicity in animals [21]. Continued investigation on the root extract of this plant led to detection of enzyme having strong fibrinolytic activity that differs from plasmin. Unlike plasmin, it can destabilize disordered co-aggregate of fibrin and Aβ peptide. This novel fibrinolytic component may lead to the development of new and effective molecules to address complex cerebrovascular diseases.

## Materials and Methods

### Reagents

Fine chemicals were procured as follows: BSA, DAPI, DEAE-cellulose, fibrinogen (bovine plasma, Type I-S), fibrinonectin (human plasma), HFIP, human serum, HSA, insulin, lysozyme, myoglobin, ovalbumin (chicken egg white), riboflavin, STI, S-2251, Sepharose CL-4B, thyroglobulin, thioflavin T dye, tranthyretin and dialysis tubing cellulose membrane (25 mm diameter; cut off range 12 kDa) from Sigma, USA; pre-stained protein Mw ladder for SDS-PAGE (PageRuler, 10–170 kDa) from Fermentas, USA; BCIP, NBT and Trypsin gold (mass spectrometry grade) from Promega, USA; CarboxyLink Coupling Gel from Thermo Scientific Products, USA; DMEM, fungizone, Ham’s modification of F-12, heat inactivated FBS, L-glutamine, MTT, PBS, penicillin/streptomycin and trypsin/EDTA solution from GibcoTM, Invitrogen, Paisley, USA; mica sheets (ASTM grade ruby mica, 20 x 20 mm, 0.27–0.33 mm thickness) from Mica Fab, Chennai, India; carbon coated copper grids (GSCu300C) for EM from ProSciTech, Australia; FITC-human fibrinogen, rabbit polyclonal Aβ (1–42) primary antibody, rabbit polyclonal human plasminogen antibody and LDH assay kit from Abcam, UK; Alexa fluor 633-labelled goat anti-rabbit IgG secondary antibody from Molecular Probes, USA; immobilized pH gradient (IPG) 2-D gel electrophoresis strips (7 cm, pH 3–10) from Bio-Rad, USA; HPLC grade solvents and analytical grade common reagents from SRL, Mumbai, India. Aβ42 (>95% pure) was from American Peptide Company, USA. Its monomeric state was confirmed from Protein Pak 60 Size Exclusion-HPLC column (fractionation range 1–20 kDa) where it appeared as a single peak of molecular mass around 4 kDa. In MS analysis, the peptide showed a mass of 4515.1102 Da (theoretical mass 4514.10 Da) as the only entity.

### Ethics statement

The purpose and the protocol of the animal experiment were explained to the Member—Secretary of the Institutional Animal Ethics Committee (IAEC) (Approval No. IICB/AEC-APP, dated 13/12/2011) and were approved by the Committee for the Purpose of Control and Supervision of Experiments on Animals (CPCSEA).

### Plant material

Fresh *A*. *indica* roots were procured from certified vendors and stored at -80°C. A flowering specimen was identified by the Botanical Survey of India, Sibpur, Howrah and preserved at the institute repository (No. 18/12). Preparation of the root extract using 10 mM Na-phosphate, pH 7.5 (buffer A) has been described in 21.

### Purification of enzyme

The extract (3 mg/ml, 100 ml) was applied to a DEAE-cellulose column (120 x 12 mm) pre-equilibrated with buffer A at 4°C. The unabsorbed fractions that showed the desired activity were pooled and applied to a substrate affinity column (40 x 7.5 mm) pre-equilibrated with buffer A at 4°C. The matrix for substrate affinity column was prepared by coupling fibrinogen with CNBr—activated Sepharose CL-4B resin (Sigma-Aldrich, USA) [22]. The bound fractions were eluted by application of 0–1 M NaCl gradient in the same buffer. All chromatograms were followed at 280 nm and at a flow rate of 20 ml/hr. Purity of the preparation was confirmed by 10% SDS-PAGE and Protein Pak 125 SE-HPLC monitoring at 220 and 280 nm. A Specord 200 spectrophotometer (Analytica Jena, Germany) equipped with temperature controlled system (PolyScience, USA) or a microplate spectrophotometer (BioTek-Epoch, BioTek Instruments, USA) was used for optical measurements.

### Mass analysis

For MS/MS analysis, proteins were digested with trypsin-gold (porcine), desalted (C_18_ zip-tip cartridge, Millipore) and were analyzed in a saturated solution of CHCA in 50% acetonitrile/0.1% TFA using MALDI TOF/TOF (Model 4800, Applied Biosystems, USA) instrument operating in reflectron mode [23]. The protein sequences were searched against Swissprot and NCBInr database using Mascot software (Matrix Science Ltd., London, UK). The MS/MS spectrum of the tryptic peptide of the purified plant protein was analyzed using the automatic *de novo* function of GPS Explorer™ Software version 3.6 (Applied Biosystems, USA). Peptide sequences of six or more amino acids with 60–100% confidence were matched to the NCBI nonredundant protein database using the protein BLAST algorithm (version 2.2.28).

### Amino acid sequencing

A sequencer (Model: PPSQ-31A, Shimadzu, Japan housed at Bose Institute, Kolkata) with an on-line phenylthiohydantoin (PTH) analyzer was used for N-terminal sequencing of proteins. Desalted and dried samples were dissolved in acetonitrile and spotted onto PVDF membrane which was directly subjected to sequence analysis.

### Fibrinolytic/fibrino(geno)lytic activity

White opaque fibrin gels were formed in 35 mm petridishes (Tarsons Product Pvt. Ltd., India) by polymerization of a solution of 160 mg fibrinogen fraction I and 2.4 U of thrombin in 10 ml of 70 mM ammonium sulfate at 25°C for 2 hr [24]. The plant extract (0–100 μl, 1 mg/ml in 100 μl) was applied over the gel, incubated at 37°C up to 96 hr and the volume of lysed fibrin was measured. Positive and negative controls were provided by 100 μl of PBS in presence and absence of plasmin. A standard curve was constructed correlating the volume of lysed gel *versus* units of plasmin activity applied (0–10 mU), where a linear dependency was observed (R^2^ = 0.965 where R^2^ is regression coefficient). From this standard curve the fibrinolytic activity of the *A*. *indica* root extract and purified enzyme was calculated. Degradation of fibrin was also followed using a Protein Pak 300 Å SE-HPLC column (Waters, 7.5 x 300 mm, fractionation range 10–300 kDa) pre-equilibrated with buffer A containing 100 mM NaCl at 0.8 ml/min. Elution of samples (50 μl) was monitored at 220 and 280 nm.

### Gel electrophoresis

Time course of degradation of fibrinogen was followed by 10% SDS-PAGE. In short, 200 μl of fibrinogen (10 mg/ml in buffer A) was incubated with 20 μl enzymes (1 mg/ml) at 37°C. Aliquots of 20 μl were withdrawn, held at 100°C for 5 min for inactivating the proteases and the products were analyzed. In another set, fibrinogen was incubated with the enzymes under identical conditions for 20 hr and analyzed by 10% PAGE under non denaturing conditions. Fibrin zymography was performed in 10% PAGE co-polymerized with fibrinogen (15.0 mg/ml) and thrombin (0.1 U/ml). After electrophoresis, SDS was partially removed by incubating the gels in 2.5% Triton X-100 for 1 hr at 25°C followed by incubation in 50 mM Tris—HCl, pH 7.5 containing 200 mM NaCl, 0.02 mM CaCl_2_ and 0.02% Brij-35 at 37°C for 24 hr when proteolysis of the substrate in the gel occurred [25,26]. The gels were stained with Coomassie Blue and destained. 2-D fibrin zymography was performed using 7 cm isoelectric focusing strip of pH 3–10 and 10% PAGE containing fibrinogen (15.0 mg/ml) and thrombin (0.1 U/ml) [21].

### Dot blot analysis

*A*. *indica* root extract (10 μg, 3 mU of fibrinolytic activity), plasmin (1.5 μg, 3 mU as positive control), BSA (1.5 μg) and purified snake venom L-amino acid oxidase (1.5 μg) (last two samples as negative controls) were spotted on nitrocellulose membrane strips. Dot blot analysis using rabbit polyclonal antibody to human plasminogen (1:1000 in PBS containing 0.05% Tween-20 and 1% skimmed milk) was performed [27]. The color was developed in 0.1 M Tris-HCl, pH 8.8 containing 0.02% BCIP and 0.03% NBT.

### Stability of the enzyme

Thermal stability of the plant enzymes was determined from the residual fibrinolytic activity after exposing at 4–100°C for 1 hr. Similarly, stability against pH was determined after incubating the enzymes for 1 hr at 37°C in buffers of pH 1.5–11.0. Residual activity (%) was calculated from the volume of lysed gel using plate assay where the activity of the control enzyme without pre-incubation was treated as 100%. Transverse 0–8 M urea gradient zymography gel was prepared (15% acrylamide, 0 M urea– 11% acrylamide, 8 M urea each containing 15.0 mg/ml of fibrinogen and 0.1 U/ml of thrombin) perpendicular to the direction of electrophoresis [28]. To check stability against blood proteases, the enzyme (0.1 mg/ml) was incubated with human serum (0.01 mg/ml in PBS) for 0–12 hr at 37°C. 10% fibrin zymography was done to assess their residual activities. For protease classification, the enzyme was incubated with 1 mM of protease inhibitors for 1 hr at 25°C and the residual activities were measured by the plate assay.

### Assay of plasmin and plasminogen activator

Plasmin or plasmin-like activity was assayed from the amidolysis of the substrate S-2251 (2 mg/ml in water, ~ 3.6 mM, ε_316_ nm = 1.27 x 10^4^ mol^-1^cm^-1^) [29]. The plant enzyme in (20 μg in 60 μl of buffer B, 50 mM Tris—HCl, pH 7.8 and 0.01% Tween 20) was put into a cuvette. Hydrolysis was initiated by adding 940 μl of 0.09 μM of S-2251in buffer B. Rate of formation of p-nitroaniline was followed at 405 nm and at 37°C for 3 min. One unit of plasmin activity was defined as the amount of enzyme that can increase A_405_ of 0.001/min. Plasmin (10 μg) served as positive control. The plant enzyme without substrate was used for correction of background absorbance. For assay of plasminogen-like activity, 100 μg of the plant enzyme and 60 μg of plasminogen were diluted to 300 μl of buffer B; 60 μl aliquots of which were added to 940 μl of 0.09 μM S-2251 in buffer B. The positive and negative controls were served by 60 μg of plasminogen in buffer B with or without 10 μg of urokinase.

### *In vivo* model for thrombolytic activity

Female Sprague Dawley rats (150–300 gm) were maintained in-house at 25 ± 1°C having 30% of humidity with light dark cycles of 12 hr. The animals were divided into four groups, each containing five. Blood was collected through retro orbital puncture after anesthesia. The clotted blood was repeatedly flushed to form uniform cloudy particles and injected into the vein of right foot pad of the same animal to form thrombus. After 1 hr of injection, samples (100 μl) were injected into the same place of thrombus formation and thrombolytic activity was followed by monitoring the physical changes in the treated footpads and behavioral pattern of the animals. Group one received plasmin as positive control (0.1–1.0 mg/kg), group two received the plant extract (1.0–10.0 mg/kg); group three received the purified enzyme (0.5–5.0 mg/kg) and group four received PBS as negative control. Dose administered was set from pilot experiments and approximately 100–1000 mU of enzyme was applied. Finally, the animals were euthanized by ketamine overdose (200 mg/kg).

### Preparation of soluble fibrin-Aβ42 co-aggregate

Fibrin-Aβ42 co-aggregate was formed by incubating 250 μl fibrinogen (1 mg/ml in PBS), 15 μl Aβ42 (3 mg/ml in DMSO) and 20 mU of thrombin in 1 ml of PBS at 37°C for 24 hr [1]. The co-aggregate of fibrin with other plasma and non-plasma proteins like fibronectin, HSA, lysozyme and transthyretin were formed by incubating the proteins (1 μM) with fibrinogen (1.5 μM) in presence of 100 μU/ml of thrombin in PBS at 37°C for 24 hr [13]. Aβ42 peptide (3.0 mg/ml) was dissolved in HFIP, sonicated in a water bath for 10 min, put into aliquots in polypropylene micro-centrifuge tubes and kept at 25°C until it became a clear solution. The solution was then dried under vacuum and stored at -20°C. Prior to use, the thin film of HFIP-treated Aβ42 peptides were dissolved in DMSO and diluted to 100 μM by 10 mM Na-phosphate, pH 7.5 containing 100 mM NaCl. It was incubated at 37°C with mild shaking to form soluble aggregates. Generally the soluble oligomers (trimer, tetramer etc) and long fibrils were formed when incubated at 37°C for 24 hr and 7 days respectively [30]. Formation of oligomers and aggregates were confirmed by mass spectrometry and AFM analysis before performing experiments.

### Cell culture

Human neuroblastoma cell line (SH-SY5Y) was maintained in medium containing DMEM/Ham’s modification of F-12 (1:1 v/v) supplemented with 10% FBS, 2 mM L-glutamine, 1% penicillin/streptomycin, gentamycin and 0.2% fungizone. The cells were routinely subcultured using 0.25% trypsin solution and grown at 37°C in a humidified incubator with 95% air/5% CO_2_ in tissue culture flasks.

### AFM

The morphological features of proteins were analyzed applying samples (10 μl) on to freshly cleaved mica (ASTM grade ruby mica, 20 x 20 mm, 0.27–0.33 mm thickness; Mica Fab, Chennai, India) surface and imaging was done as described [30]. For analysis of the accumulation of co-aggregates on cell surface and its degradation by enzyme, the morphology of SH-SY5Y cells was analyzed in AFM. The cells were seeded on to glass cover slips at a density of 10^6^ cells/ml in a 6-well plate. The cells were treated with the test samples, *viz*., fibrin-Aβ42 co-aggregate preincubated with or without the plant enzyme or plasmin for 24 hr at 37°C. An equal volume of medium was added to control cultures and the cells were incubated for another 48 hr at 37°C. The analysis was performed using a Pico plus 5500 AFM (Agilent Technologies, USA) instrument. For imaging of protein samples a 9 μm piezoscanner and micro fabricated silicon cantilevers (225 μm in length) with a nominal spring force constant of 21–98 N/m were used. Cantilever oscillation frequency was tuned into 150–300 kHz resonance frequency. Imaging of cellular samples was done in liquid mode using 100 μm scanner and cantilevers of 450 μm length with a nominal spring force constant of 0.2 N/m. The resonance frequency was set at 13 kHz. Picoview 1.10.1(9995) software was used for image analysis.

### SEM

Protein samples (10 μl) were placed on a carbon coated grid (GSCu300C; ProSciTech, Australia) for 5 min at 25°C and the unbound portion was removed by a blotting paper. To stain the adhered particles, the grid was treated with 2% uranyl acetate for 20 sec and the excess reagent was removed as stated. The grid was dried under vacuum, sputter coated with gold-palladium alloy and viewed under SEM. The imaging was done using SEM (model Vega II LSU, Tescan Digital Microscopy Imaging, Czechoslovakia) at 10.0 kV voltage.

### Confocal microscopy

FITC—conjugated human fibrinogen (5.2 mg/ml), Aβ42 (0.05 mg/ml) and thrombin (20 mU/ml) were incubated in 1 ml of PBS at 37°C for 24 hr with mild shaking. FITC conjugated fibrin-Aβ42 was preincubated for 24 hr with plasmin as positive control, the purified enzyme as test samples and PBS as negative control. Thereafter, the samples were diluted with fresh medium, added to the SH-SY5Y cells (10^6^ cells/ml) and incubated additionally for 48 hr at 37°C. The cover slips were washed twice with PBS, fixed for 20 min using 4% formaldehyde at 25°C and the nonspecific sites were blocked by 1% BSA for 1 hr. The co-aggregate was detected using rabbit polyclonal Aβ (1–42) antibody (1:200 dilution) followed by Alexa fluor 633-labelled goat anti-rabbit IgG (1:500 dilution). The nucleus was counterstained with 0.01% DAPI. The confocal images were collected using Andor spinning disc confocal microscope (Yokogawa CSU-X1) equipped with Andor ixon3 897 EMCCD camera and Olympus ix81 microscope (Olympus America Inc., USA).

### MTT and LDH release assays

Preformed fibrin-Aβ42 co-aggregate (10 μl) was preincubated for 24 hr with plasmin (0.2–1.0 mU), purified enzyme (0.2–1.0 mU) and PBS, diluted with fresh medium and added to 96-well polystyrene plates (Corning Inc., Corning, NY) containing 10^5^ cells/100 μl. The same volume of medium was added to cultures serving as controls. The plates were then incubated for 48 hr at 37°C. The cytotoxicity assays (MTT and LDH assay) were performed as described [30]. Cells where no MTT was added, served as negative control. The difference of absorbance was considered as 100% viability of cells [31]. The LDH activity was calculated as: LDH activity (mU/ml) = Amount of NADH in nmol x sample dilution / time interval x sample volume.

### Statistical analysis

Statistical analyses were based on at least five independent series of experiments with triplets. Unpaired *t*-tests were used for statistical analysis. Data (mean ± S.D.) were considered to be statistically significant when the two-tailed probability (*p*) value was <0.05. The datasets were analyzed in http://www.physics.csbsju.edu/stats/t-test.html.

## Results

### *Aristolochia indica* contains multiple fibrinolytic enzymes

**Fig 1:**
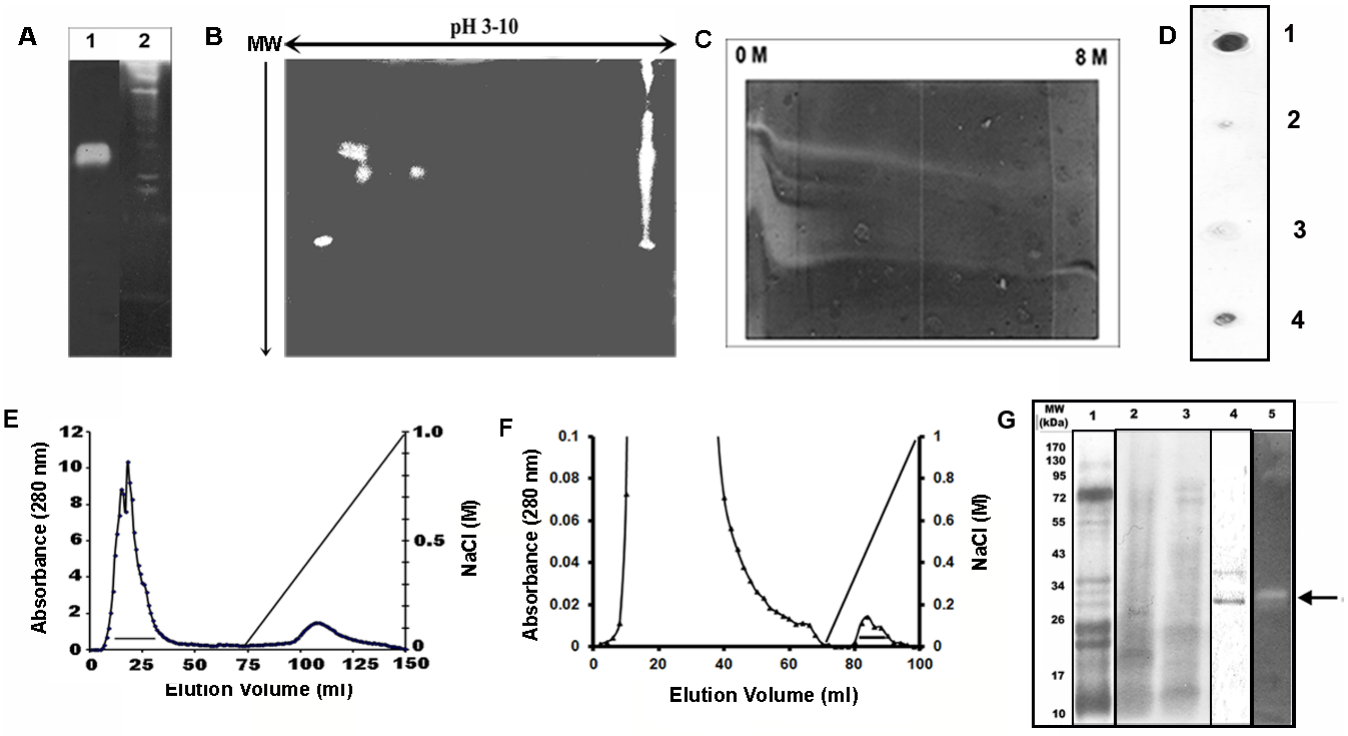
Detection and purification of fibrinolytic component from the aqueous extract of the root of *Aristolochia indica*. **(A)** fibrin zymography. Lane 1, plant extract (35.5 μg); lane 2, plasmin (5 μg). The lanes were selected from two different zymograms run under identical conditions. **(B)** 2-D fibrin zymography of the root extract (350 μg) showing the presence of multiple components; 5 spots in the acidic zone and one trailing lane in the alkaline zone. **(C)** Transverse 0–8 M urea gradient fibrin zymography showing presence of multiple enzymes which are stable up to variable denaturant concentrations. **(D)** dot blot analysis of 1, plasmin; 2, BSA and 3, Russell’s viper venom L-amino acid oxidase; 4, *A*. *indica* root extract developed against rabbit polyclonal human plasminogen antibody. **(E)** DEAE cellulose chromatography of the crude extract where a linear gradient of 0–1 M NaCl was applied. The bar represents the active fractions that were pooled; **(F)** substrate affinity chromatogram of the pooled fractions from DEAE-chromatography. The bound fractions were eluted by the application of 0-1M NaCl and the bar represents the active fractions. **(G)** SDS-PAGE of the plant extract (lane 1), unabsorbed fraction from DEAE cellulose column (lane 2), unabsorbed fraction from substrate affinity column (lane 3), absorbed fraction from substrate affinity column (lane 4) and fibrin zymography of the same sample (lane 5). The arrow indicates position of fibrinolysis. The position of the marker proteins is indicated at the left.

An aqueous extract of the root of *A*. *indica* turned opaque fibrin gels into colorless solutions in a time (24–96 hr) and concentration (0–300 μg/ml) dependent manner. As compared to plasmin, the extract contained 105±11.8 mU/mg of fibrinolytic activity. Fibrin zymography of the extract in 1-D and 2-D zymography showed existence of multiple fibrinolytic components. Compared to plasmin, these enzymes were of both higher and lower Mw (Fig 1A and 1B). From the 2-D zymography and chromatographic patterns of the extract (described later), it appeared that the alkaline enzymes dominated the pool. These fibrinolytic enzymes showed different degrees of stability against denaturation by urea in 0–8 M transverse urea gradient fibrin zymography (Fig 1C). Presence of plasmin(ogen) in the extract was checked from dot blot analysis against plasminogen antibody. Plasmin showed a distinct spot whereas, the plant extract showed faint spot indicating low abundance of protein/s having similar antigenic determinants as of plasminogen. The negative controls showed no cross reactivity (Fig 1D). Since, the plant extract showed presence of plasmin(ogen), immuno-affinity chromatography using plasminogen antibody was chosen for purification of the fibrinolytic enzymes. The extract was first applied to a DEAE-cellulose column at pH 7.5 where majority of the activity was eluted as unabsorbed fraction. The absorbed fractions also showed activity but its recovery was poor (Fig 1E). This step rendered the recovered activity colorless as the pigments present in the extract were adsorbed by the matrix. Subsequently, the unbound fraction was applied to an immuno-affinity column where the active components were eluted as unabsorbed fraction (result not shown). Plasmin(ogen) like enzyme which was eluted as the bound fraction had no fibrinolytic property. Thus, to purify the active fraction, the unabsorbed part collected from DEAE cellulose chromatography was applied to a fibrinogen coupled—Sepharose 4B substrate affinity column where majority of the protein components was eluted as unabsorbed fraction. The activity was recovered as two partially resolved fractions after application of 0–1 M NaCl gradient (Fig 1F). Presence of fibrinolytic activity in the fractions was verified by fibrin plate assay and fibrin zymography.

The purified fraction showed two distinct bands of 36.7 and 31.3 kDa in SDS-PAGE (Fig 1G). This fraction (60 ml) was lyophilized and redissolved in buffer A at 1 mg/ml. It also generated colorless liquid by lysing of opaque fibrin gels in a time (24–48 hr) and concentration (0–5 μg) dependent manner (result not shown) and had 200 mU/mg of fibrinolytic activity. Considering initial activity of the plant extract (300 mg, 26,295 mU) and recovery of 17,000 mU, yield was 65%. This pooled fraction was further separated by Protein Pak 125 Å SE-HPLC column, where two distinct components of retention time 16.31 ± 0.01 and 13.91 ± 0.09 min were eluted. The active protein of 31.3 kDa revealed partial amino acid sequence on MS/MS analysis and showed similarity with a few plant and human proteins with low score. This protein might be unique in sequence and may not have matches with database entries. Its N-terminal sequence was ‘QSEQAQQQNNNGNRG’. This showed significant identity (82%) with peptidase M23 family of *Ruminococcus sp*. and also with other proteins, most of which are either hypothetical or functionally dissimilar.

### Stability of the enzyme

Hereafter, the results shown are of the enzyme purified by the substrate affinity column. The enzyme retained full activity at 4°C and pH 7.5 for 1 hr, however, the residual activity gradually decreased under identical conditions at elevated temperature, *e*.*g*., at 90°C, residual activity was 28.7 ± 8.1%. The enzyme remained reasonably stable in the pH range of 6.5–8.0, showed maximum stability at pH 7.5 when incubated for 1 hr and at 37°C. Stability of the fibrinolytic enzyme in presence of blood proteases was verified by fibrin zymography. With respect to a control sample devoid of serum, no detectable difference of band intensities of the test samples were observed, which indicates the stability of the enzyme against blood proteases (result not shown). The activity was strongly inhibited by the metallo-protease inhibitors like EDTA and 1,10-phenanthroline, but in presence of 2 mM of Ca^+2^ and Mg^+2^ the activity was restored. Probably there are roles of metal ions in maintaining the structure and function of the enzyme. Inhibition of the activity by other cysteine and serine protease inhibitors was not significant (Table 1).

**Table 1:** Effect of protease inhibitors.

Class of inhibitor	Name of protease inhibitor	% Residual activity of enzyme^a^
**Cysteine protease inhibitor**	Iodoacetamide E-64 Na-tetrathionate	72.2 ± 0.1 95.3 ± 1.2 78.5 ± 1.4
**Serine protease inhibitor**	PMSF Benzamidine Aprotinin	99.2 ± 0.3 88.9 ± 2.1 93.4 ± 1.4
**Metalloprotease inhibitor**	EDTA 1,10-phenanthroline	6.5 ± 1.1 13.5 ± 2.5

^**a**^ The residual activity of the inhibitor treated sample was calculated with respect to the activity of enzyme in absence of any inhibitor which was considered as 100%. The concentrations of protease inhibitors were 1 mM for all the sets. The values represent mean ± S.D., where *p* < 0.05.

### The mechanism of fibrinolysis by the plant enzyme is different from plasmin

**Fig 2:**
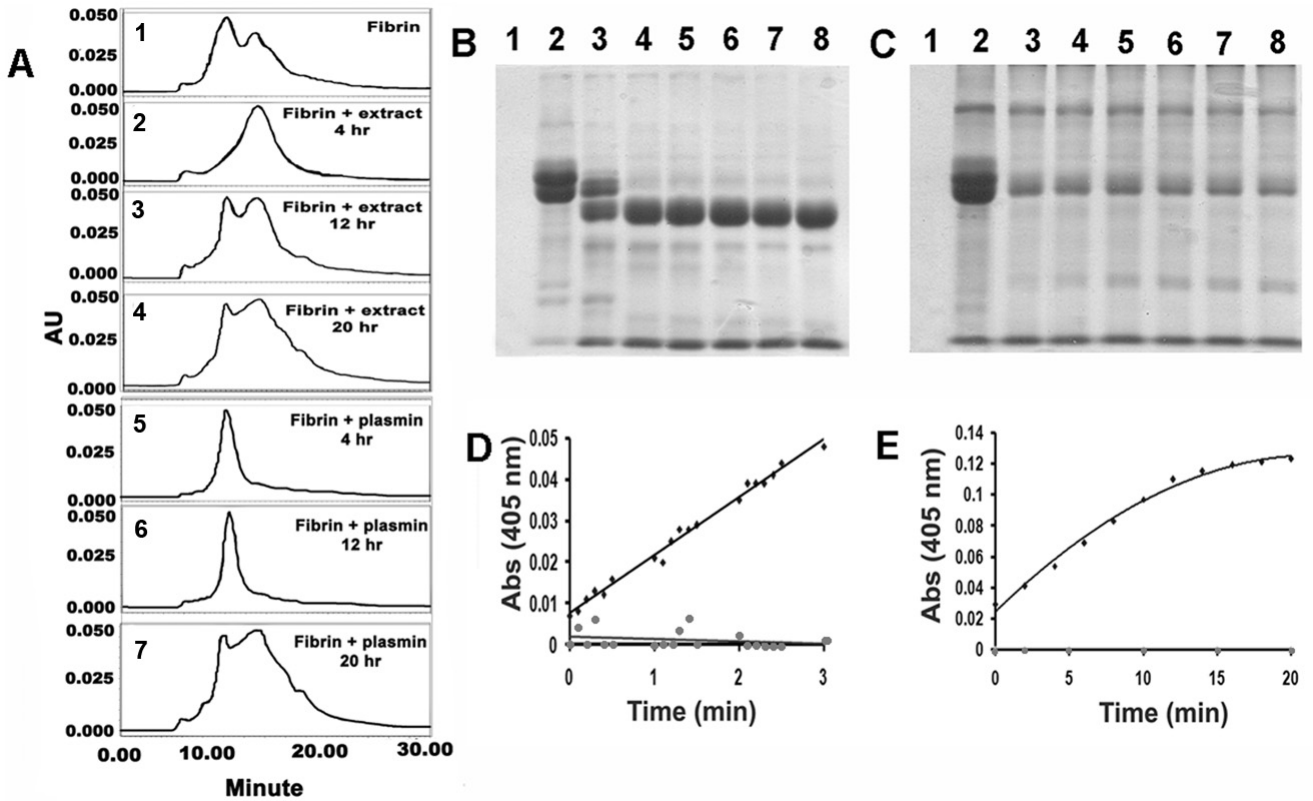
Difference in fibrinolytic properties of plant enzyme and plasmin. **(A)** Time course of degradation of fibrin by the plant enzymes and plasmin as observed from SE-HPLC. 1: Fibrin as control (10 μg); 2–4: fibrin (10 μg) treated with the plant extract (0.1 μg) for 4, 12 and 20 hr. 5–7: fibrin under identical conditions of incubation with plasmin (0.1 μg) for 4, 12 and 20 hr. A Protein Pak 300 SW column equilibrated with 10 mM Na-Phosphate, pH 7.5 containing 100 mM NaCl and at a flow rate of 0.8 ml/min was used. In all sets, the elution was followed at 280 nm. **(B-C)** time course of degradation of fibrin by the plant enzymes and plasmin as observed in SDS-PAGE. **(B)** lane 1, plant extract (0.01 μg); lanes 2–8, fibrinogen (10 μg) treated with the plant extract (0.01 μg) for 0, 1, 2, 3, 4, 5 and 6 hr respectively. **(C)** lane 1, plasmin (0.01 μg); lanes 2–8, fibrinogen (10 μg) treated with plasmin (0.01 μg) for 0, 1, 2, 3, 4, 5 and 6 hr respectively. **(D)** Time course of amidolysis of S-2251 (0.08 μM) in presence of plasmin (10 μg) (■) and plant enzyme (20 μg) (●); **(E)** Amidolytic assay for plasminogen-like activity of the plant extract (100 μg) in presence plasminogen (60 μg) (●). Plasminogen (60 μg) in presence of urokinase (10 μg) (■) served as control. Spontaneous hydrolysis of S-2251 was insignificant and has not been included to ensure clarity.

Plasmin initiates cleavage of fibrinogen and soluble fibrin from the C-terminal end of it’s α-polypeptide chain and forms fibrin degradation products (FDPs) in plasma known as fragments X, Y, D and E [32]. The SE-HPLC profiles of fibrinolysis by the plant enzyme and plasmin for 20 hr were almost similar where complete degradation of fibrin was evident. However, corresponding profiles for 4 and 12 hr were significantly different. The plant enzyme initially produced larger fragments. Subsequently, they were converted into smaller ones which were poorly resolved in HPLC. Degradation of fibrin by plasmin was not clear up to 12 hr as the products had similar retention times as of fibrin (Fig 2A). The plant enzyme completely degraded all subunits of fibrinogen to smaller fragments as demonstrated in SDS-PAGE. In contrast, plasmin produced stable fragments of defined size that did not produce smaller fragments in course of time (1–6 hr). Complete degradation of fibrinogen was prominent in case of the plant enzyme (Fig 2B and 2C).

Taken together, these observations suggested marked difference between plasmin and the plant enzyme. For further validation, ability of the plant enzyme to hydrolyze S-2251, a synthetic substrate specific for plasmin was investigated. Plasmin showed hydrolysis of S-2251 at 405 nm while the absorbance retained at basal level when the plant enzyme was used (Fig 2D). Using the same assay system, plasminogen activator-like property of the plant enzyme was verified. In one set, plasminogen was treated with urokinase, a known plasminogen activator that converts plasminogen to plasmin. Formation of plasmin was quantified by the hydrolysis of S-2251. When the same reaction was followed replacing urokinase with the plant enzyme, plasmin was not formed as the reaction mixture was incapable of hydrolyzing the substrate S-2251(Fig 2E). These indicated that the mechanisms of fibrinolysis by the two classes of enzymes were different.

### Thrombolytic activity of the enzyme *in vivo*

**Fig 3:**
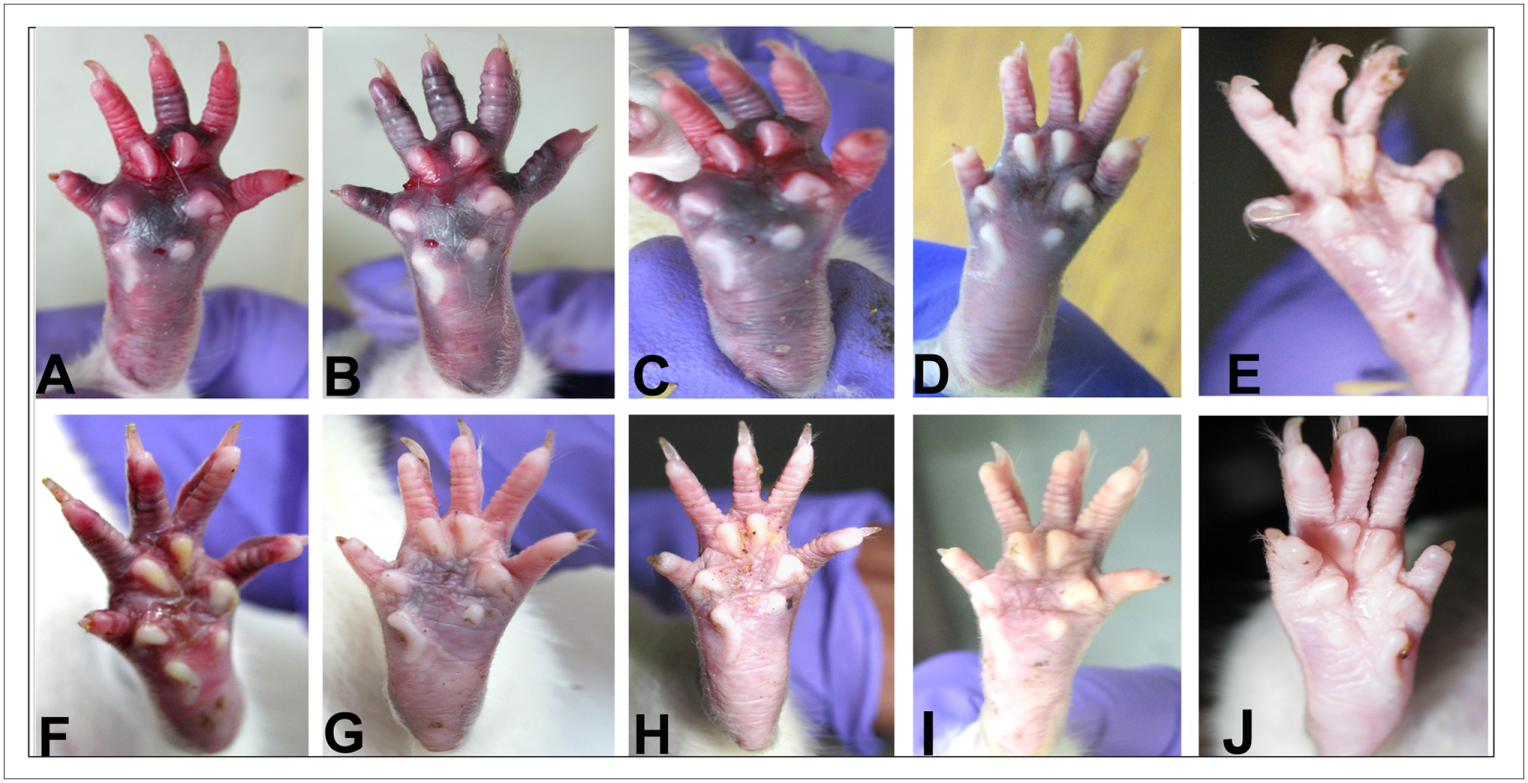
Recovery of thrombosis at rat footpad by the plant fibrinolytic enzymes. **(A-D)**, after generation of thrombus as described in the text, the following components was applied at 0 hr, **(A)** PBS (100 μl); **(B)** plant extract (10 mg/kg); **(C)** purified enzyme (5 mg/kg) and **(D)** plasmin (1 mg/kg). In **(E)**, plant extract was applied where no thrombus was generated beforehand to serve as a control to check inherent thrombolytic activity of the extract. Conditions of the corresponding set after 24 h have been illustrated in **(F-J)**. While F shows little recovery out of natural healing, **(J)** demonstrates that the plant extract is free from thrombolytic activity. Variable degrees of recoveries are evident in **(G-I)**.

To verify whether the plant fibrinolytic enzyme was capable of dissolving thrombus, blood clots were generated in the right foot pad of rats. Swelling of the pads with blue-red coloration and impairment of movement of the animals ensured thrombus generation. The animals were followed for 24 hr when it was observed that the group treated with PBS showed minimum recovery, the group treated with the plant extract showed moderate recovery, whereas the groups treated with the purified enzyme and plasmin showed good recovery. The plant enzyme when administered at 2.0–5.0 mg/kg, the rats showed recovery within 24 hr. The recovery was qualitatively estimated from disappearance of swelling and coloration of foot pads and mobility of the animals. Injection of the plant enzymes alone in the footpad did not cause any physical or behavioral change in the animals (Fig 3).

### Plant enzyme degrades fibrin-Aβ42 co-aggregate *in vitro* and *ex vivo*

**Fig 4:**
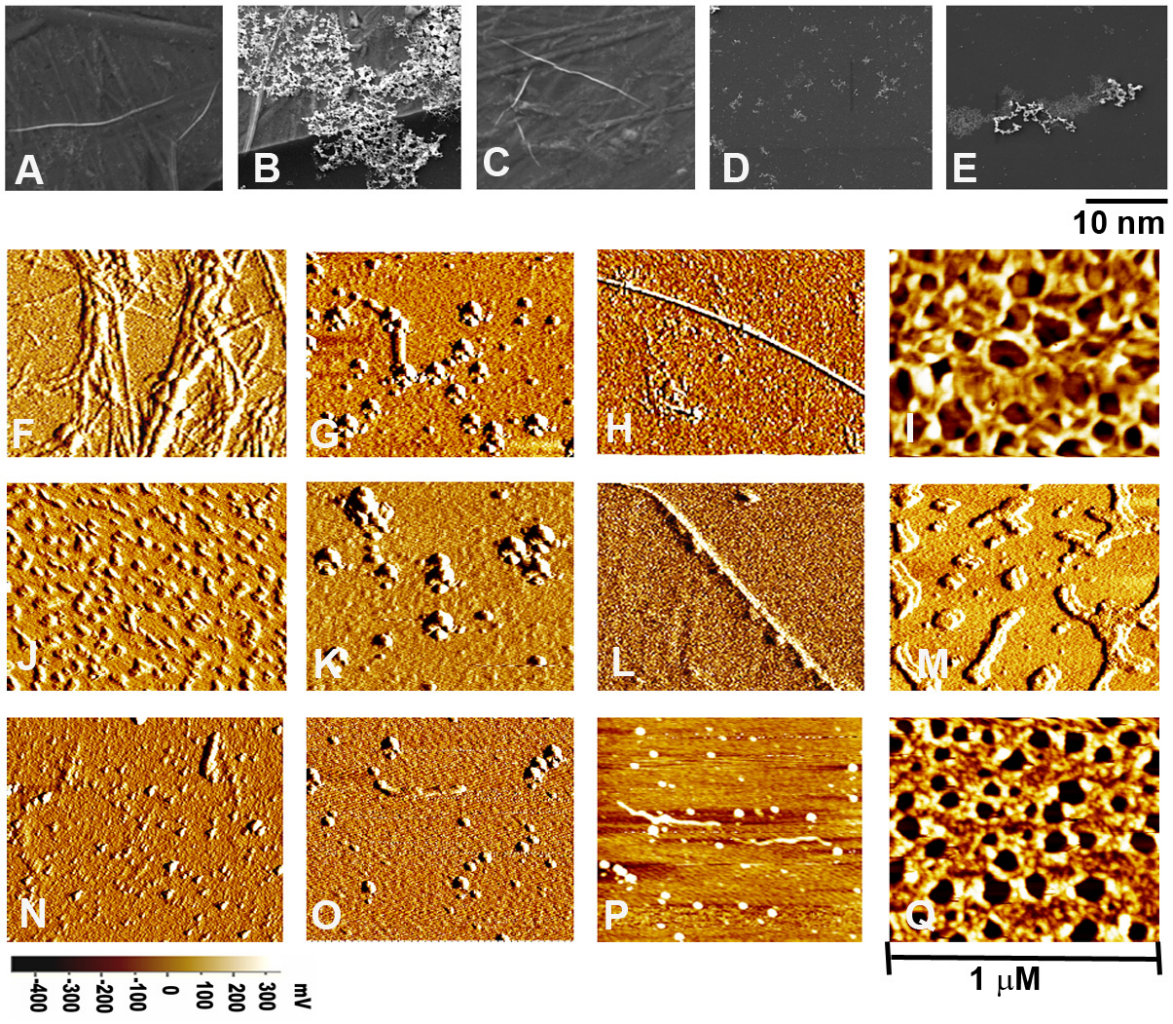
Effect of plant enzyme on morphology of Aβ42 aggregates and fibrin-Aβ42 co-aggregate. **(A)** Aβ42 (20 μM) fibrillar form obtained after incubation of Aβ42 peptide (100 μM DMSO solution) in 10 mM sodium phosphate, pH 7.5, containing 100mM NaCl at 37°C with mild shaking for 7 days; **(B)** fibrin-Aβ42 co-aggregate showing denser and heavily cross-linked structure; **(C)** preformed Aβ42 aggregate (20 μM) after incubation for 24 hr in presence of the plant enzyme showing presence of intact fibrils; **(D)** preformed co-aggregate after incubation for 24 hr in presence of the plant enzyme showing complete degradation of the structure leading to monomeric and very small oligomeric forms; **(E)** preformed co-aggregate incubated for 24 hr in presence of plasmin showing fragmented residual structures that are large, compact and cross-linked. The resolution and magnification 10 nm and 30,000X respectively. **(F)** fibrin network; **(G)** Aβ42 oligomer (20 μM) obtained after incubation of Aβ42 peptide (100 μM DMSO solution) in 10 mM sodium phosphate, pH 7.5, containing 100mM NaCl at 37°C with mild shaking for 24 hr **(H)** Aβ42 aggregate (20 μM); **(I)** fibrin-Aβ42 co-aggregate; **(J)** fibrin treated with plant enzyme showing smaller globular fragments; **(K)** Aβ42 oligomer treated with plant enzyme showing presence of oligomeric forms; **(L)** Aβ42 aggregate after incubation for 24 hr in presence of the plant enzyme retain its fibriliar structure; **(M)** the co-aggregate treated with plant enzyme showing random fragmentation of the fibrillar structure, **(N)** Fibrin treated with plasmin; **(O)** Aβ42 oligomer treated with plasmin showing smaller monomeric forms; **(P)** Aβ42 aggregate treated with plasmin for 24 hr showing few fragments along with small monomers; **(Q)** co-aggregate pretreated with plasmin showing partial trimming of the structures maintaining thick fibrillar network. The horizontal bar shows the amplitude (mV) of the images. The scan size was 1 μm.

Since the mechanisms of fibrinolysis of plasmin and the plant enzyme were different and plasmin was sluggish in destabilizing fibrin—Aβ42 co-aggregate, we explored whether the plant enzyme could effectively act on the co-aggregate. The structural features of the aggregates were followed by SEM and AFM. The SEM image of Aβ42 aggregate (20 μM) showed presence of fibrillar structure (Fig 4A), whereas fibrinogen (0.6 μM) in presence of Aβ42 (2.0 μM) formed a dense irregular network (Fig 4B). Plant enzyme (10 μl, 1.0 mU) was unable to destabilize the fibrils of Aβ42 (20 μM) when incubated at 37°C for 24 hr (Fig 4C). In fact, prolonged incubation of Aβ42 aggregate with the plant enzyme didn’t show significant differences (result not shown). The dense co-aggregate (50 μl) was completely degraded by the plant enzyme (10 μl, 1.0 mU) to form small particles of 1–2 nm diameter that corresponded to monomer/dimer and not of higher oligomers (Fig 4D). Under identical conditions, plasmin (10 μl, 1.0 mU) partially degraded the dense co-aggregates leading to residual loose aggregates. With increase of incubation period or the amount of plasmin, these structures remained unaltered (Fig 4E).

Corresponding AFM images at higher resolution supported these observations. Upon polymerization by thrombin, fibrinogen monomers of diameter 70 nm formed regular fibrillar network of fibrin of width 20–50 nm (Fig 4F). Aβ42 forms oligomeric species when incubated at 37°C for 24 hr (Fig 4G). On the other hand, Aβ42 formed fibril of average width of 45 nm when incubated at 37°C for 7 days (Fig 4H). Fibrinogen copolymerized with Aβ42 formed completely different thick and dense irregular structures having small globular aggregates (36–47 nm width) attached to the network (Fig 4I). Plant enzyme showed strong fibrinolytic activity by degrading the fibrin network into small globular fragments (Fig 4J). It couldn’t degrade the Aβ42 oligomers or aggregate efficiently as revealed from Fig 4K and 4L, where oligomeric forms and long fibrils along with small bead-like molecules were apparent. The plant enzyme degraded the irregular network of co-aggregate to form loosely associated small uniform fragments of diameter 24–45 nm (Fig 4M). Plasmin degraded the fibrin, Aβ42 oligomers and fibrils efficiently (Fig 4N–4P). However, plasmin could at most degrade the dense network to smaller superficial fibrils (Fig 4Q). These results encouraged to study the effect of the plant enzyme in inhibiting localization of co-aggregate on SH-SY5Y human neuroblastoma cells.

**Fig 5:**
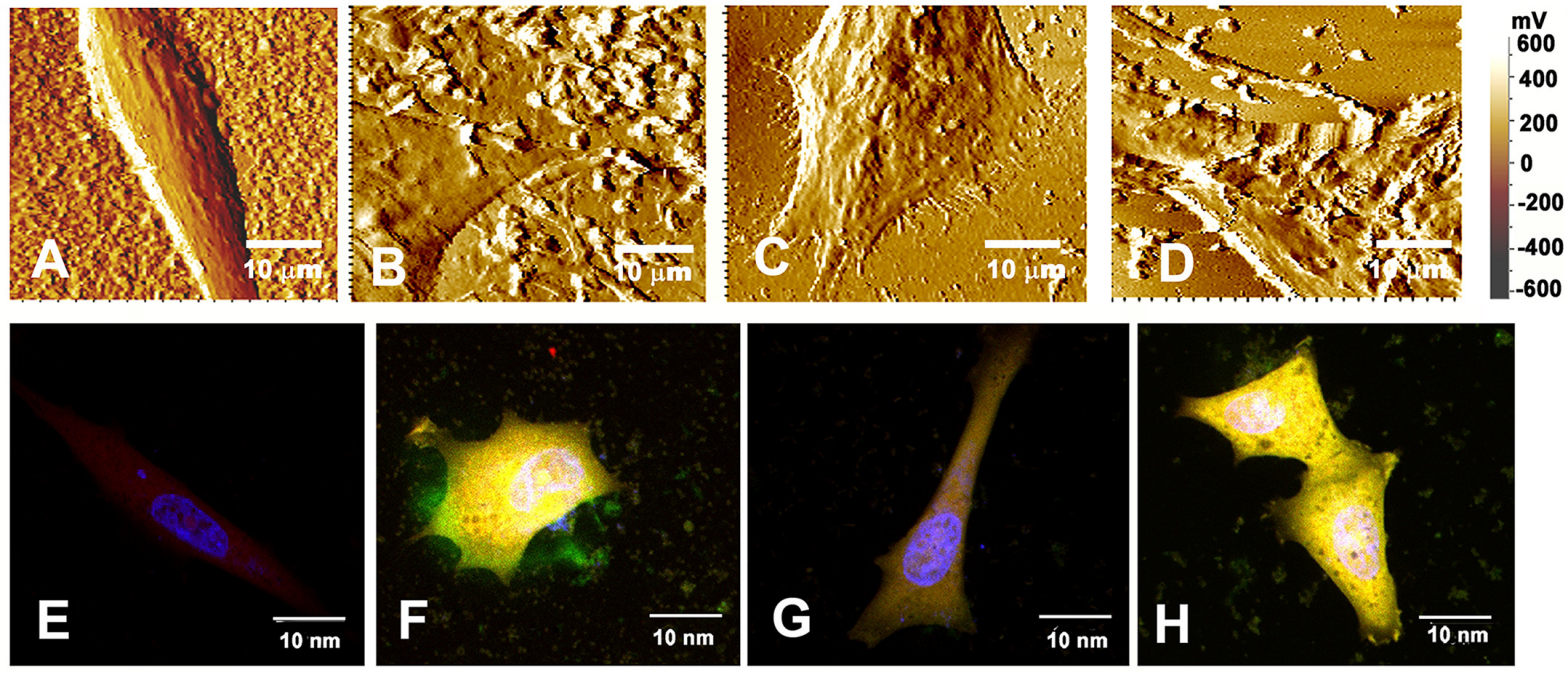
Inhibitory effect of plant enzyme on interaction of fibrin-Aβ42 co-aggregate with neuroblastoma cells **(A)** Untreated cells; **(B)** deposition of preformed fibrin-Aβ42 co-aggregates on cell surface after incubation for 48 hr at 37°C; **(C)** removal of the co-aggregates from the cell surface after incubation with the plant enzyme; **(D)** residues of fibrin-Aβ42 co-aggregates on the cell surface after incubation with plasmin. The resolutions of images E-K were variable according to the area of selection to get a clear presentable image. **(E)** untreated cells with elongated morphology showing prominent blue nuclei (DAPI stained) and very faint expression of Aβ42 appearing as red as Alexa fluor 633 conjugated anti-rabbit secondary antibody was used against rabbit polyclonal antibody to Aβ1–42 peptide; **(F)** cells treated with fibrin-Aβ42 co-aggregate showing intense greenish-yellow fluorescence (as human fibrinogen conjugated with FITC showing green fluorescence and Aβ42 showing red fluorescence colocalized) indicating penetration of the co-aggregate inside the cells and deposition on the extracellular surface. The cells have markedly different morphology from untreated cells. **(G)** Cells treated with co-aggregate preincubated with plant enzyme showing morphology and absence of localization of the co-aggregate similar to that of the untreated cells as of **(E)**; **(H)** cells treated with co-aggregate preincubated with plasmin showing failure of prevention of its localization within the cells and deposition on the cell surface. The morphology also differs from the untreated cells. The magnification of all images was 60X and resolution was 10 nm.

AFM and confocal imaging were employed to examine deposition of fibrin-Aβ peptide co-aggregate on neuroblastoma cells and their clearance by the plant enzyme in comparison to plasmin. The topography of the untreated SH-SY5Y cells in AFM showed a smooth and clear surface (Fig 5A). Cells incubated for 48 hr with preformed fibrin-Aβ42 co-aggregate (50 μl) generated from 6 μM fibrinogen and 20 μM Aβ42 revealed appearance of clumps on its surface (Fig 5B). In its continuation, the preformed co-aggregate was treated separately with 1.0 mU of the plant enzyme or plasmin for 24 hr and the supposedly degraded products were incubated with the cells for additional 48 hr. The cells treated with the co-aggregate and the plant enzyme didn’t show presence of deposits of the co-aggregate on the cell surface and the cells showed healthy condition with its stretched processes (Fig 5C). In contrast, plasmin treated co-aggregate was unable to prevent deposition of the clumps of the co-aggregates on the cell surface (Fig 5D).

The soluble oligomers of Aβ42 were localized within the perinuclear cytosol and extracellular space to exert cytotoxicity [33]. For this, immuno-cytochemical analysis was performed by incubating the neuroblastoma cells for 48 hr at 37°C with fibrin-Aβ42 co-aggregate preincubated with or without the plant enzyme (1.0 mU) or plasmin (1.0 mU). The confocal image of the untreated cells showed faint expression of intrinsic Aβ42 peptide as indicated by very low emission of red fluorescence spread over the body of the cell (Fig 5E). The co-aggregate treated cells were in degenerating condition, showed localization of the co-aggregate inside the cells and extracellular surface as indicated by intense greenish-yellow fluorescence (Fig 5F). When the co-aggregate preincubated with the plant enzyme was applied, the cells showed similar characteristics as untreated cells with very low emission of red and undetectable greenish yellow fluorescence. This indicated that the plant enzyme might convert the co-aggregate into smaller fragments that were unable to deposit on the cell surface, enter into the cells and exert toxicity (Fig 5G). Plasmin, in contrast, was ineffective in removing deposition of co-aggregate from the cell surface (Fig 5H). AFM and confocal images collectively indicated that the plant enzyme was more efficient than plasmin in degrading the co-aggregate and reduce its toxicity in neuroblastoma cells. The confocal images were more conclusive in this respect as it demonstrated that the residual structures of the co-aggregate treated with plasmin could still penetrate the cells whereas the plant enzyme yielded such entities that apparently did not interact with the cells, therefore the question of toxicity did not arise.

### The plant enzyme reduces co-aggregate induced cytotoxicity

**Fig 6:**
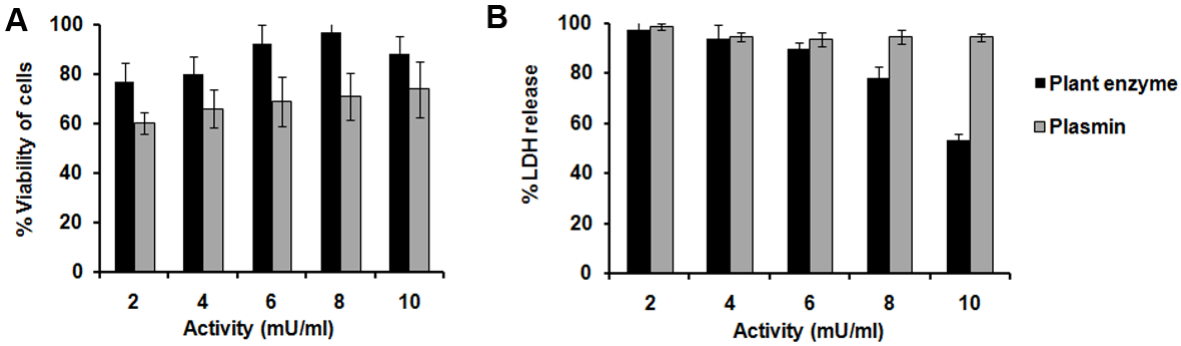
Reduction of co-aggregate induced cytotoxicity by the plant enzymes. **(A)** MTT assay: viability of cells was plotted against the concentration of plant enzyme (black bar) and plasmin (gray bar). The viability of the untreated cells was considered as 100%. **(B)** LDH assay. The % of LDH released from cells was plotted against the concentration of plant enzyme (black bar) and plasmin (gray bar). LDH released from the co-aggregate treated cells was considered as 100%. The values of the untreated cells were subtracted from the test samples. The bars represent mean ± S.D. of five independent experiments in each set. Probability values of *p* < 0.05 were considered to represent significant differences. The probability values *(p* > 0.05) of viability and % LDH release from cells treated with co-aggregate were compared with cells treated with co-aggregates preincubated with plasmin. Insignificant difference between these two groups was observed.

The fibrinolytic enzyme showed better inhibition of co-aggregate induced cytotoxicity on SH-SY5Y cells in a dose dependent manner as compared to plasmin. In MTT assay, the cells treated with co-aggregate (50 μl) generated from 6 μM fibrinogen and 20 μM Aβ42, showed 56.2 ± 5.9% viability. Viability of the cells were preserved up to 97.0 ± 4.2% when the cell were treated with co-aggregate that was preincubated with the plant enzyme (0.8 mU). On the other hand, plasmin (0.2–1.0 mU) showed only 10–15% increase in the cell viability (Fig 6A). Cell viability was correlated with LDH assay, where increase of LDH activity of cultured cells was indicative of cytotoxicity due to release of LDH into the medium upon cell damage or lysis. In this assay, LDH release from the co-aggregate treated cells was considered as 100% assuming exertion of maximum toxicity. When the co-aggregate preincubated with the plant enzyme (0.2–1.0 mU) was applied to the cells, release of LDH decreased significantly. Co-aggregate induced LDH release was reduced to nearly 50% by the plant enzyme (1.0 mU). In contrast, 1.0 mU of plasmin showed only 6% reduction in LDH release (Fig 6B). The plant enzyme or plasmin even at the highest concentration applied here did not increase the release of LDH as compared to control untreated cells (result not shown). Therefore, the plant enzyme having 0.8 mU activity was sufficient for inhibiting co-aggregate induced cytotoxicity.

### Degradation of various fibrin co-aggregates by the plant enzyme

**Fig 7:**
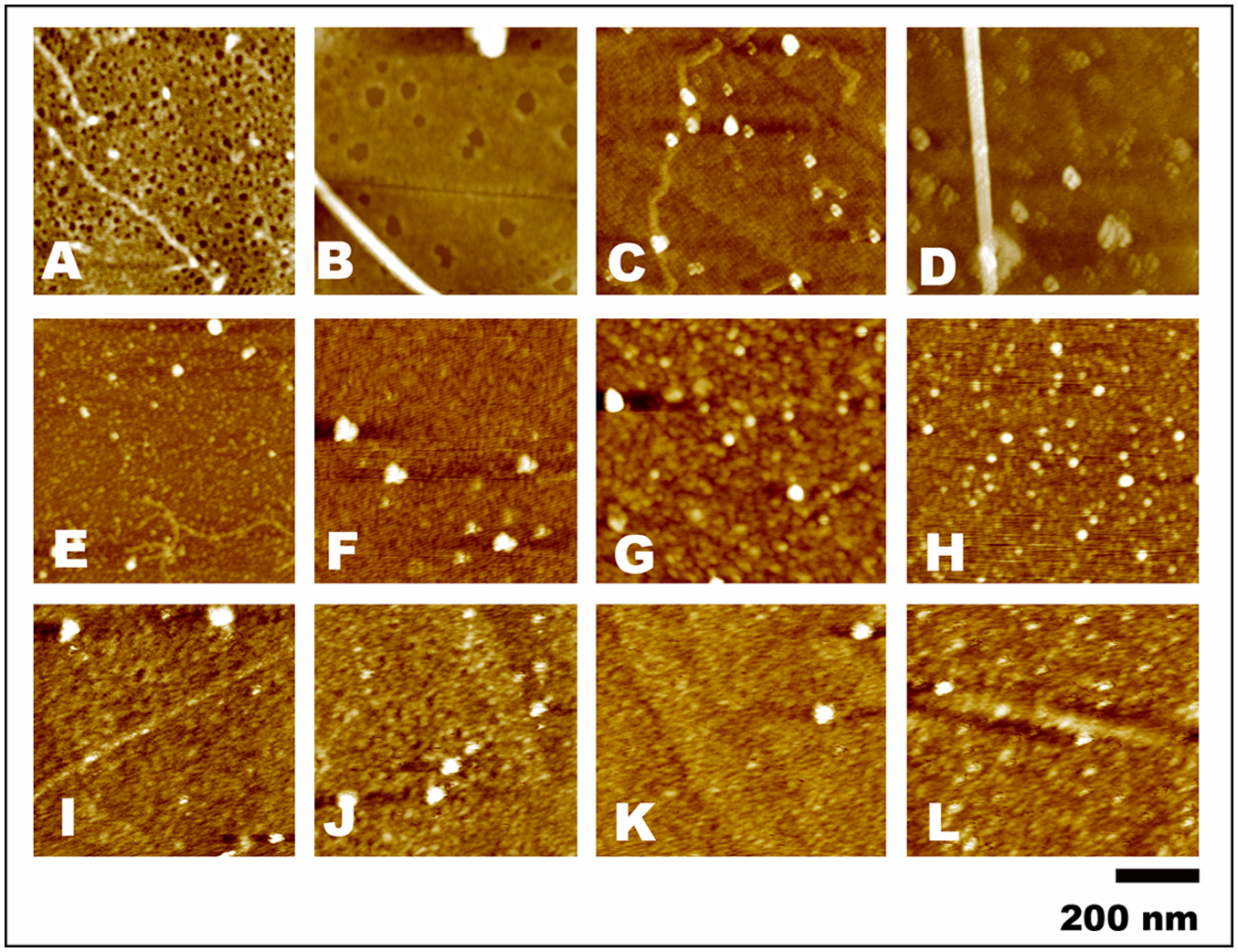
Topographic AFM images of fibrin-plasma protein co-aggregates. **(A-D)** co-aggregates of fibrin-HSA, fibrin-lysozyme, fibrin-transthyretin and fibrin-fibronectin respectively. **(E-H)** These co-aggregates were treated with plant enzyme for 24 hr and their corresponding morphological features were shown. **(I-L)** The morphology of the co-aggregates treated with plasmin for 24 hr are illustrated. Features have been described in the text. The instrumentation was same as described in Fig 5.

Experiments stated above encouraged investigating whether the plant enzyme was also capable of degrading other fibrin-plasma protein co-aggregates over plasmin. Among plasma and non-plasma proteins that form aggregates and cause serious pathological consequences, fibronectin, albumin, lysozyme and tranthyretin were selected for preparing fibrin co-aggregates. Fibrin-HSA co-aggregate formed dense honey-comb like structure with numerous small pores along with long fibrils (Fig 7A). Fibrin-lysozyme co-aggregate also formed similar structures, but the pores were bigger (Fig 7B). Fibrin-transthyretin and fibrin-fibronectin co-aggregates have dense network with prominent long fibrils (Fig 7C and 7D). The plant enzyme significantly degraded these co-aggregates to different extents in 24 hr. Degradation of fibrin-HSA co-aggregate was lesser than the fibrin-lysozyme co-aggregate where smaller fragments of fibrin-transthyretin and fibrin-fibronectin were formed (Fig 7E and 7H). Plasmin could not degrade the dense networks or fibrils of fibrin-plasma protein co-aggregates (Fig 7I and 7L). Therefore, the plant enzyme degraded not only the fibrin-Aβ42 co-aggregate, but also other abnormal clots to a significant extent. This observation strengthens the broad specificity of the plant enzyme towards degradation of various abnormal fibrin clots.

## Discussion

Involvement of proteases and their regulation are intricately involved in all phases of wound healing. Dissolution of fibrin clot is important to maintain fluidity of blood after damaged tissue repairing [34,35]. An aqueous extract of the root of the plant *A*. *indica* showed presence of multiple fibrinolytic enzymes. Such enzyme in plant has evolutionary significance [36–39]. They might be evolved in *A*. *indica* as a host defense mechanism. Plant defense molecules often represent a cornucopia of effective therapeutic compounds for human diseases. A simple *in vivo* animal model avoiding complicated, expensive and invasive procedures for assessment of thrombolysis was developed. It demonstrated rapid healing property of the plant enzyme that was comparable to plasmin [40,41]. However, the substrate specificity of the plant enzyme differed from plasmin. It is expected because of wide phylogenic difference between their sources. There are very limited reports on fibrinolytic enzymes, the mechanisms of which differ from that of plasmin or plasmin-like enzymes. Effects of these enzymes on destabilizing other type of protein aggregates have not been worked out [42].

Abnormal fibrin clots are usually resistant to proteolysis by plasmin and lead to serious pathological complications [13]. The plant fibrinolytic enzyme being functionally different from plasmin, its ability to dissolve abnormal fibrin clots, especially fibrin-Aβ co-aggregate was investigated. Monitoring destabilization of abnormal aggregate by enzymes using the dye Thioflavin T (ThT) showed inconsistent results. In fact, turbidometric analysis also showed inconsistencies. These might be due to the presence of partially degraded aggregates variable sizes in the solution. Thus quantification of the extent of destabilization of abnormal aggregates by the plant enzymes was difficult. However, microscopic analysis showed prominent changes in the physical structure of the aggregates. The plant enzyme degraded dense network of the fibrils to smaller fragments more efficiently than plasmin. Exogenously applied oligomeric Aβ has been shown to interact with lipid raft and accumulate on the neuronal surface [43]. The exogenously applied fibrin-Aβ co-aggregate also formed network on the neuronal surface and induced stress that causes morphological and functional changes of neuron. It is also localized inside the neuron causing toxicity. Pre-incubation of fibrin-Aβ co-aggregate with plant enzyme interfered with the formation of the fibrillar network and formed small non toxic fragments that did not form network on cell surface and the cells showed healthy morphology unlike the co-aggregate treated cells. Talens *et al* identified eighteen fibrin clot-bound proteins [44]. They have pathological significance as these proteins aggregate and cause considerable damage to the respective organs where they are deposited; *e*.*g*., aggregation of fibronectin and lysozyme cause multiple sclerosis and hereditary systemic amyloidosis respectively [45,46]. Transthyretin is associated with several pathologies such as senile systemic amyloidosis (SSA), familial amyloid polyneuropathy (FAP) and familial amyloid cardiomyopathy (FAC) which are characterized by extracellular deposition of insoluble amyloid fibrils [47]. Therefore, efficiency of the plant enzyme in degrading these fibrin-plasma protein co-aggregates was also verified. Results showed that these co-aggregates are better lysed by the plant enzymes as compared to plasmin though the efficiency varies.

**Fig 8:**
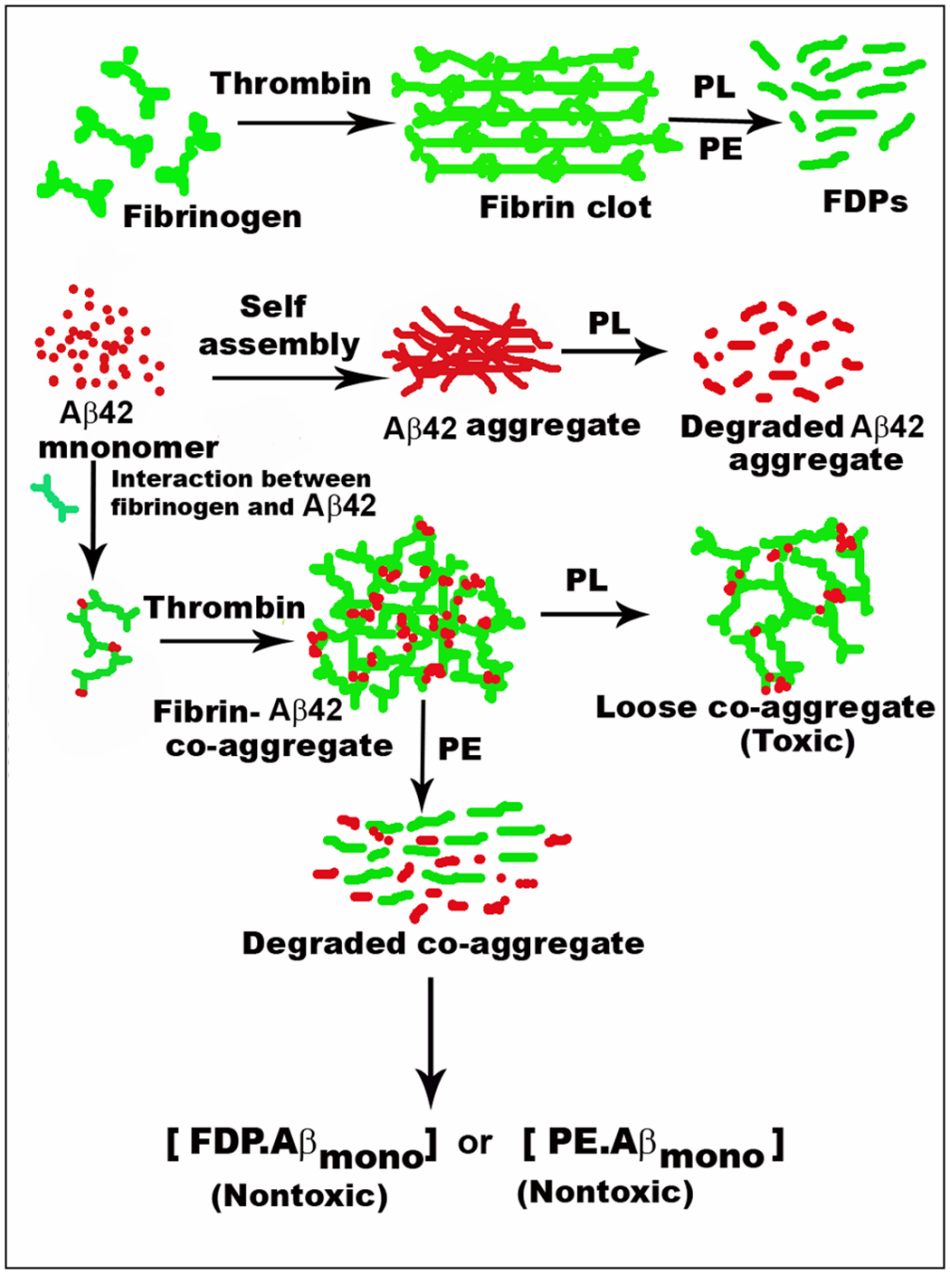
Hypothetical model of fibrin-Aβ42 co-aggregate formation and its destabilization by the plant enzymes (PE) or plasmin (PL). Fibrinogen and Aβ42 form aggregates independently that are degraded by PL. PE also degrades fibrin clot. When fibrinogen interacts with Aβ42, it forms abnormal co-aggregate. The efficiency of PE is superior to PL in destabilizing the co-aggregate. FDP stands for fibrin degradation products. Stable end products are marked bold.

The experimental evidences give an idea about the mechanism by which the neuronal cells are protected from the toxicity exerted by the fibrin—Aβ co-aggregate. It has been verified separately that the Aβ peptide in monomeric, oligomeric or fibrillar state were stable against the plant fibrinolytic enzyme. Therefore, in the fibrin—Aβ co-aggregate, fibrin was the target of proteolytic degradation and its susceptibility was not reduced in the co-aggregate structure. It is agreed that in the co-aggregate, the molecularity of Aβ peptide remains unknown; it may exist as a monomer or short oligomers but not in a fibrillar state because the co-aggregate never yielded residual fibrillar structures upon fibrinolysis. What is noteworthy, these monomeric or oligomeric forms of Aβ peptides generated from the destabilized fibrin co-aggregate did not show toxicity on neuroblastoma cells. Therefore, the released amyloid peptides did not appear to be present in its free native like state or conformation to follow their natural propagation towards aggregation manifesting cellular toxicity. Considering the consequences of the course of the reactions, the amyloid peptides are likely to remain bound to fibrin fragments. Of course, one can not exclude binding of the peptides with the plant enzymes or their auto-digested products, if any, but this possibility is rather remote as the abundance of the degraded components of fibrin far superseded the enzyme. Proposed reaction scheme of plasmin *versus* the plant enzyme is described in Fig 8. In summary, the plant enzyme could be useful for designing better therapeutic agents for diseases caused by deposition of protease resistant heterogeneous fibrin clots.

## Supporting Information

S1 ARRIVE Checklist NC3Rs ARRIVE Guidelines Checklist. (DOCX)

## Abbreviations

AFM: atomic force microscopy
BCIP: 5-Bromo-4-chloro-3-indolyl phosphate
CHCA: α-cyano-4-hydroxycinnamic acid
CNBr: cyanogen bromide
DAPI: 4',6-diamidino-2-phenylindole
DMEM: Dulbecco's modified eagle medium
DMSO: dimethylsulfoxide
FITC: fluorescein isothiocyanate
HFIP: 1,1,1,3,3,3-hexa-fluoro iso-propanol
HSA: human serum albumin
LDH: lactate dehydrogenase
MTT: (3-[4,5-dimethylthiazol-2-yl]-2,5-diphenyltetrazolium bromide)
NBT: Nitro blue tetrazolium
PBS: phosphate buffered saline, pH 7.2
SE-HPLC: size exclusion high performance liquid chromatography
SEM: scanning electron microscopy
STI: soyabean trypsin inhibitor
S-2251: H-D-valyl-L-leucyl-L-lysine-p-nitroaniline dihydrochloride
TFA: trifluoroacetic acid
NMDA: *N-*methyl-D-aspartate. 1 or 2-D in electrophoresis refers to 1 or 2-dimensional
