# Supplementary material for: An Enzyme from Aristolochia indica Destabilizes Fibrin-β Amyloid Co-Aggregate: Implication in Cerebrovascular Diseases
Source: PLoS One. 2015 Nov 6;10(11):e0141986. doi: 10.1371/journal.pone.0141986 (PMC4636252; doi:10.1371/journal.pone.0141986)

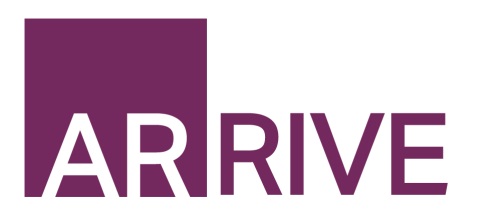


The ARRIVE Guidelines Checklist

Animal Research: Reporting In Vivo Experiments

Carol Kilkenny^1^, William J Browne^2^, Innes C Cuthill^3^, Michael Emerson^4^ and Douglas G Altman^5^

*^1^The National Centre for the Replacement, Refinement and Reduction of Animals in Research, London, UK, ^2^School of Veterinary Science, University of Bristol, Bristol, UK, ^3^School of Biological Sciences, University of Bristol, Bristol, UK, ^4^National Heart and Lung Institute, Imperial College London, UK, ^5^Centre for Statistics in Medicine, University of Oxford, Oxford, UK.*

|  | | ITEM | RECOMMENDATION | Section/ Paragraph |
| --- | --- | --- | --- | --- |
| 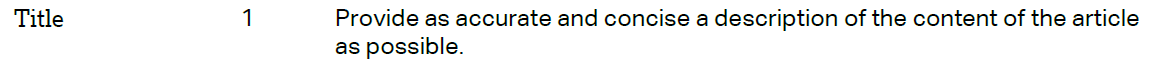 | | | Title |  |
| 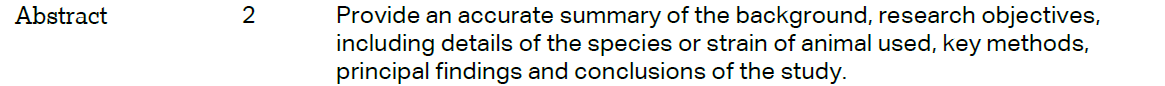 | | | Abstract |  |
| INTRODUCTION | | |  |  |
| 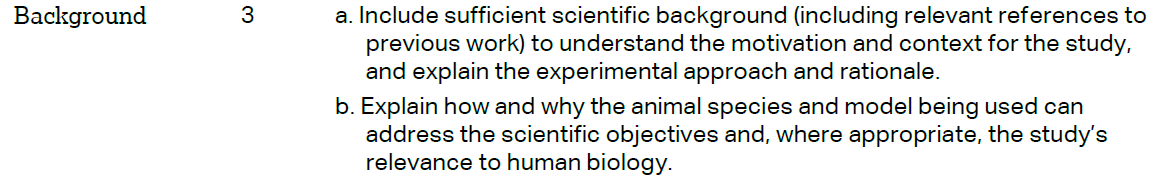 | | | IntroductionThroughout |  |
| 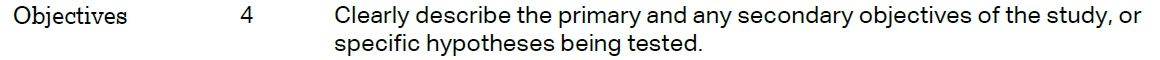 | | | Introduction Paragraph 4 |  |
| METHODS | | |  |  |
| 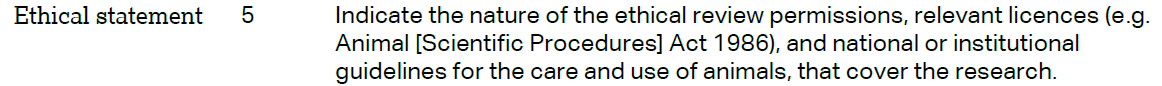 | | | Ethics statement |  |
| 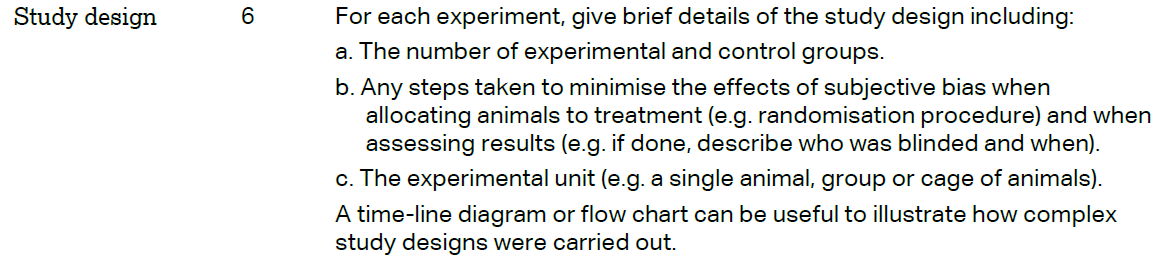 | | | In vivo model for thrombolytic activity |  |
| 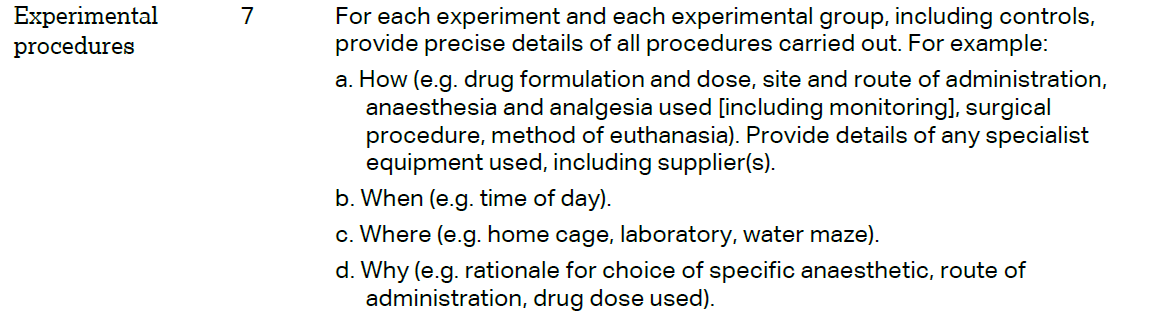 | | | In vivo model for thrombolytic activity |  |
| 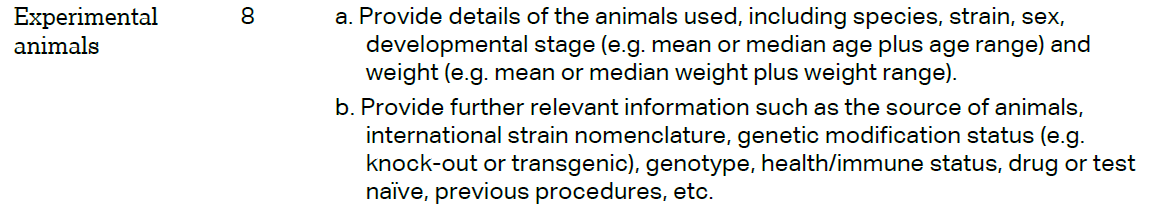 | | | In vivo model for thrombolytic activity |  |

The ARRIVE guidelines. Originally published in *PLoS Biology*, June 2010^1^

| 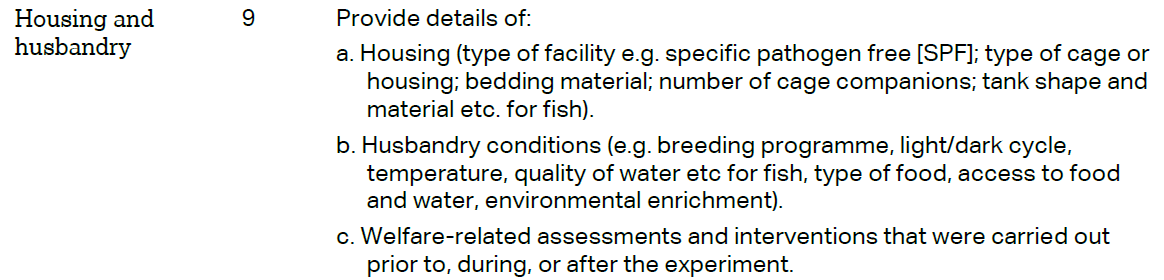 | In vivo model for thrombolytic activity | |
| --- | --- | --- |
| 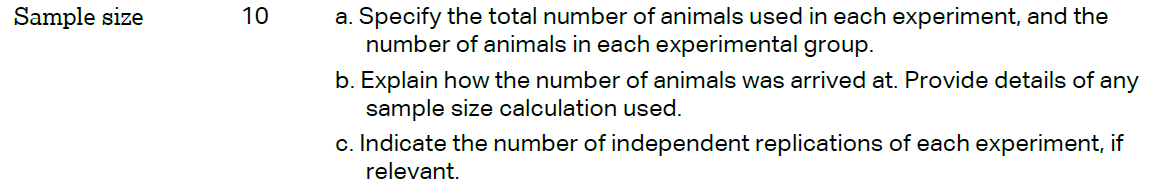 | In vivo model for thrombolytic activity | |
| 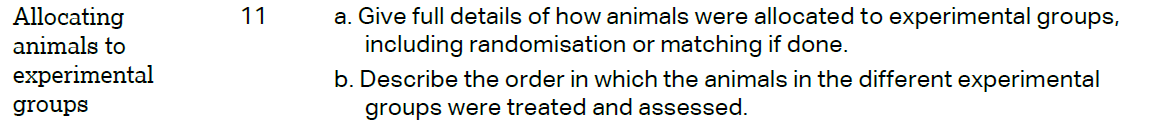 | In vivo model for thrombolytic activity | |
| 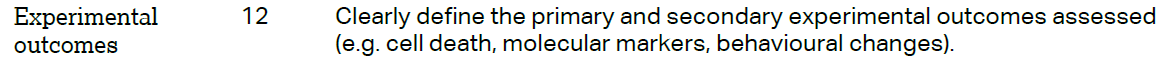 | Result  Thrombolytic activity of the enzyme in vivo | |
| 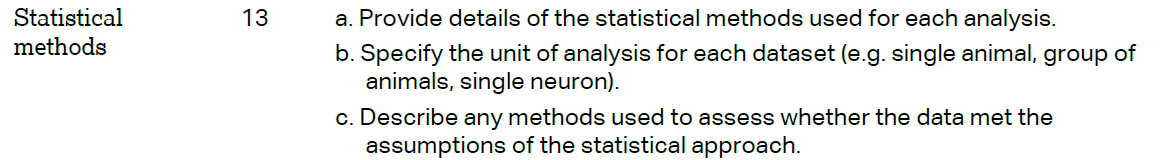 | Statistical analysis | |
| RESULTS |  | |
| 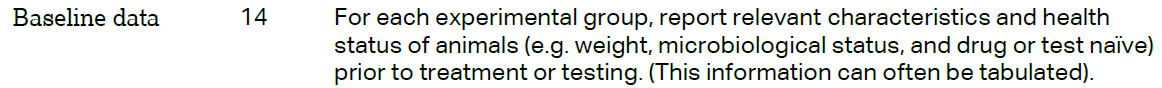 | Thrombolytic activity of the enzyme in vivo | |
| 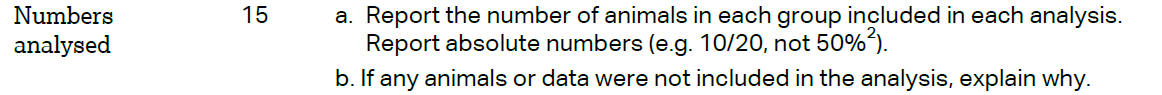 | Thrombolytic activity of the enzyme in vivo | |
| 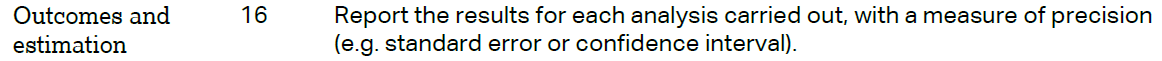 | Thrombolytic activity of the enzyme in vivo  Figure 3 | |
| 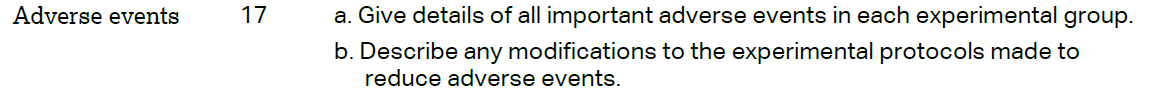 | Thrombolytic activity of the enzyme in vivo | |
| DISCUSSION |  | |
| 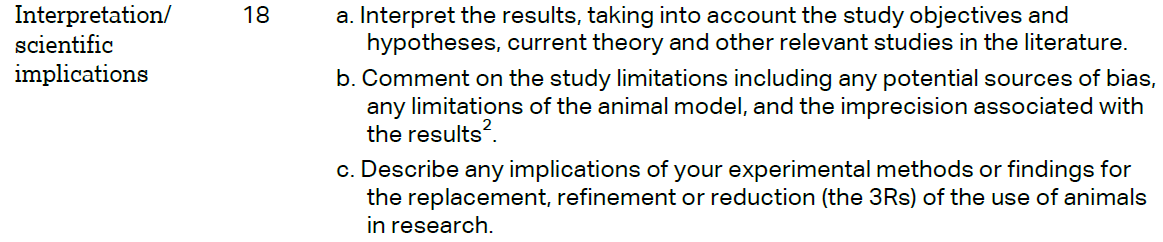 | Paragraph 1-3. | |
| 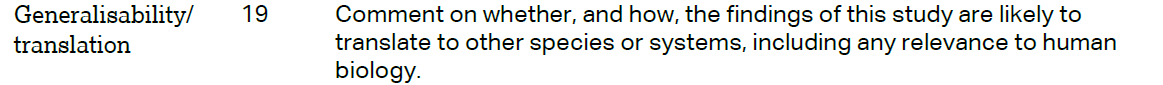 | Paragraph 3 | |
| 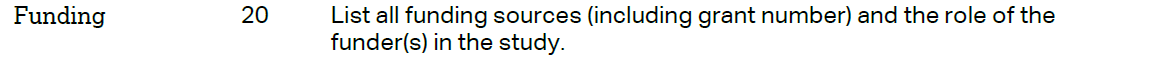 | | Funding |


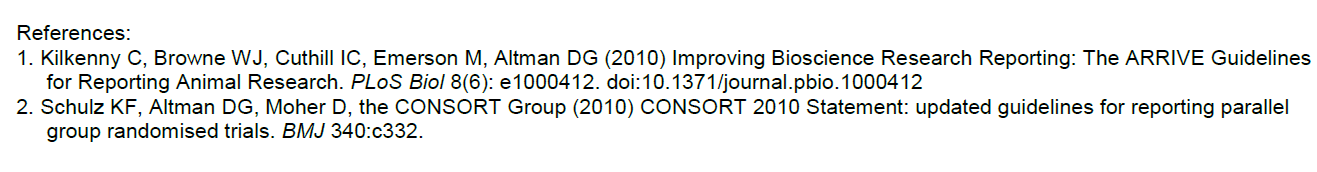

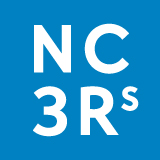

Supplement: S1 ARRIVE Checklist — (DOCX) [file pone.0141986.s001.docx]
